# Navigating the Research Landscape of Emotional and Social Intelligence Among Young Adults: A Bibliometric Perspective

**DOI:** 10.7759/cureus.59130

**Published:** 2024-04-27

**Authors:** Binny Jose, Angel Thomas

**Affiliations:** 1 Psychology, Lincoln University College, Kuala Lumpur, MYS; 2 Department of Health and Wellness, Marian College Kuttikkanam, Kuttikkanam, IND; 3 Psychology, Mar Sleeva Medicity, Palai, Kottayam, IND

**Keywords:** biblioshiny., vosviewer, young adults, social intelligence, emotional intelligence, bibliometric analysis

## Abstract

This bibliometric analysis examines the research landscape on emotional and social intelligence in young adults, highlighting its impact on both personal and professional spheres, including overall wellness and happiness. Understanding the current research status in this area is crucial for identifying existing knowledge gaps, emerging trends, and possible future research and application directions. Utilizing bibliometric techniques, the study evaluates numerous scholarly articles from the Scopus database, employing tools like VOSviewer and Biblioshiny for data analysis. The research scope covers articles published from 1990 to 2023 across multiple disciplines such as psychology, education, sociology, and management, focusing on key bibliometric indicators like publication trends, citation patterns, prominent institutions and authors, and thematic clusters. The findings indicate a growing interest among youth in emotional and social intelligence over the past three decades, as demonstrated by the increasing volume of literature. This growing interest underscores the significance of this topic in modern research. By mapping the academic network and collaborative efforts in the field, the study identifies leading contributors whose work has significantly advanced understanding in this area. The insights gained could help shape future research endeavors, inform policy-making, and aid educators in incorporating emotional and social intelligence into educational programs to support the holistic development and well-being of young people. The research also identifies key authors, key journals, key institutions, and possible collaboration and publication opportunities. Using thematic mapping and keyword patterns, some emerging trends are brought to light with the prime objective of guiding future studies and initiatives.

## Introduction and background

Emotional and social intelligence play crucial roles in the personal and social development of young adults. As they transition from adolescence to adulthood, young adults encounter new challenges, such as forging meaningful relationships, managing their emotions effectively, and navigating complex social interactions. They are going through a period of confusion, both inwardly and outwardly [1].

The ability to identify, comprehend, and handle one's own emotions, as well as properly react to others' emotions, is referred to as emotional intelligence (EI) [2]. According to Brasseur et al. [3], it consists of self-awareness, self-control, drive, empathy, and social abilities. Social intelligence is the capacity to perceive, comprehend, and regulate social relationships. It is similar to emotional intelligence. This includes the ability to establish and sustain connections as well as work well with others [4].

It is vital for young adults to comprehend the ideas of emotional and social intelligence and how they apply to them. The studies show that social intelligence, emotional intelligence, and cultural intelligence are closely connected [5]. Later studies proved that emotional and social intelligence has an impact on a variety of life domains, including self-esteem, interpersonal connections, social skills, critical thinking, creative thinking, academic success, job success, and fulfilling interpersonal relationships [6]. In addition, developing young people's emotional and social intelligence can lead to favourable societal outcomes [7], including fewer disputes, improved empathy, higher prosocial behaviour, and a happier existence [8,9]. 

Using this bibliometric evaluation, we aim to enhance the body of knowledge on emotional and social intelligence in young adults, assisting researchers, practitioners, and policymakers in their initiatives to support emotional health, constructive social interactions, and individual development during this crucial stage of life. This study intends to aid in creating evidence-based treatments, programmes, and strategies that promote the emotional and social development of young people and eventually improve their general well-being and success by identifying gaps, trends, and new research areas.

A potent software programme for visualising and examining bibliometric networks is called VOSviewer. It gives academics a complete set of tools to browse and interpret massive amounts of bibliographic data. VOSviewer allows people to find patterns, correlations, and developments in academic literature owing to its user-friendly interface and sophisticated visualisation capabilities [10,11]. The "bibliometrix" R package's user interface is referred to as "Biblioshiny." An R package called "Bibliometrix" was created particularly for bibliometric analysis. It offers a wide range of tools and features for processing and displaying bibliographic information. Researchers without a lot of programming knowledge can utilise the "Biblioshiny" component of the package, which is an interactive user interface that enables users to do bibliometric studies using a graphical interface. Users can easily import their bibliographic information into "Biblioshiny," preprocess the information, and perform a number of bibliometric studies, such as co-authorship analysis, citation analysis, and keyword co-occurrence analysis.

## Review

Review of literature

Throughout history, various definitions of intelligence have been used. There have been a variety of definitions, from Pythagoras' explanation of intelligence as "winds" to Descartes' understanding of intelligence as the ability to distinguish between true and false [12]. 

The ability to comprehend, control, and navigate one's own emotions, as well as successfully engage in interpersonal relationships with others, is referred to as emotional and social intelligence [13]. EI, in the view of Brasseur et al., comprises being conscious of one's feelings, understanding them, and being aware of how feelings may influence decisions. You may lessen your tension, express yourself effectively, empathise with people, get beyond difficulties, and settle disputes as a result of it. Understanding emotion itself can help one improve emotional intelligence [14]. Even though the study of emotional intelligence is an emerging discipline, Salovey and Mayer coined the phrase in 1990 [15] in a piece of literature. It also entails the ability to manage and control one's own emotions with the goal of adapting effectively to different circumstances and handling pressure. The capacity to understand and navigate social dynamics, interpret nonverbal cues, and effectively communicate are all examples of social intelligence. It necessitates skills like empathy, attentive listening, assuming different viewpoints, and conflict resolution.

The concept of EI has gained attention in recent years. There are different models of assessing and interpreting emotional intelligence. The emotional competence inventory (ECI) incorporates self-assessment and others' assessments, which try to provide a 360° perspective [16]. The ability model, proposed by Mayer and Salovey, understood “emotional intelligence as the ability to perceive, appraise, and express emotion; to use emotions to facilitate thinking; to understand emotions; and to regulate emotions for growth. Recent studies show that emotional intelligence is closely associated with subjective well-being [17].

Sundvik and Davis studied the role of emotional intelligence in handling social media stress and mental health. They concluded that emotional intelligence can reduce the possibility of social media stress. The study also stated that emotional confidence can help with mental health [18].

Theorists have provided a wide range of definitions of social intelligence, but they all have two things in common: their understanding and reaction to others, and how they adjust to social settings. “Social intelligence helps an individual develop healthy coexistence with other people. Socially intelligent people behave tactfully and prosper in life. Social intelligence is useful in solving the problems of social life and helps in tackling various social tasks” [19].

According to several research studies, social intelligence is complex and different from general intelligence domains. According to Carr and Hancock [20], these conceptions of social intelligence take into account both internal and external perceptions, social skills, and other psychosocial characteristics. The study by Marlowe speaks of five domains of social intelligence, namely prosocial attitude, social skills, empathy skills, emotionality, and social anxiety [21]. The study by Saxena and Kumar [22] discussed the social intelligence of undergraduate students, examining the influence of gender and subject stream. The study reveals that female students exhibit higher social intelligence compared to male students, and arts students have higher social intelligence than science students. The study emphasises the importance of social intelligence in managing personal life and interpersonal relationships.

Ford and Tisak [23] discussed the concept of social intelligence and its relationship to academic intelligence. The study used various measures to assess social intelligence and found that it is indeed a separate factor. The study makes recommendations for social cognition and competency research, as well as for social skills and education initiatives.

Swain discusses the social and emotional challenges faced by adolescents during their transition from childhood to adulthood. The study examines characteristics of adolescence, such as moodiness, emotional tension, and restlessness, and addresses various issues that adolescents encounter, including physical growth, mental competition, emotional disturbances, home and sex adjustment, vocational problems, student activism, use of alcohol and drugs, quarrels, impatient behaviour, and peer group influence. The article concludes by suggesting that adolescents require social and emotional support from adults to navigate through this challenging period [24]. Zakirova and Irina discuss the relationship between the success of training activities and social intelligence. The study involved 140 students and evaluated the success of training and social intelligence using data from the last session. The article emphasises the importance of social intelligence in effective interpersonal interaction and successful social adaptation [25].

Anwar et al. studied the relationship between emotional intelligence, attachment style, and social intelligence and concluded that individuals with secure attachment styles had higher levels of emotional and social intelligence, while those with insecure attachment styles had lower levels. Social intelligence was found to moderate the relationship between insecure attachment styles and emotional intelligence. The study suggests that attachment styles and emotional intelligence play important roles in understanding social relationships [26]. The study by Louise Cherry Wilkinson on Social Intelligence and the Development of Communicative Competency reveals the importance of social intelligence and advocates for the inclusion of social and personal factors in understanding human problem-solving behaviour [27]. With a particular focus on students, Avlaev investigated the function of social intelligence in the formation of the self. It explores the dynamics, purposes, and interactional levels of social intelligence. The idea of maturity is also examined, encompassing many sorts like ego maturity, psychological maturity, psychosocial maturity, and psychosocial maturity. The paper includes study findings on the association between various facets of social intelligence as well as the relationship between social intelligence and maturity. The result highlights the significance of social intelligence in a person's total maturity, especially in a learning environment. In earlier studies, self-awareness was considered to be the basis of social intelligence [28]. The study by Gulliford et al. proved that social intelligence can be developed in an individual. Their study put forward gratitude and self-monitoring as ways to build social intelligence [29].

Relevance of the study

The bibliometric study on "social intelligence and emotional intelligence in young adults" is highly relevant as it systematically analyses and quantifies the impact and trends of research in this vital area. The study can identify key contributors and influential works by examining the number of publications, citations, and collaborations, providing valuable insights for policymakers, educators, and researchers. Understanding the dynamics of social and emotional intelligence in young adults can contribute to developing effective educational and psychological interventions, ultimately promoting healthier social interactions and emotional well-being in this critical demographic.

Materials and methods

We procured scientific publications relevant to our investigation from the primary collection of the Scopus database. On July 21, 2023, we conducted a search using specific keywords such as "Emotional Intelligence," "Social Intelligence," and "Young Adults." Our search was not limited by language and focused solely on articles, excluding book chapters and reviews. In total, we gathered 917 articles from 397 different sources, spanning the years 1998 to 2023. To ensure accuracy, we screened the Scopus records to eliminate duplicates. The results were then saved as a "CSV" file. For analysis, we employed VOSviewer version 1.6.19 and Bibloshiny software to perform bibliometric analysis on the collected data [30]. Table 1 provides comprehensive information about the critical aspects of our investigation.

**Table 1:** Critical aspects of the investigation.

Description	Results
Search query	(TITLE-ABS-KEY ("Emotional Intelligence") OR TITLE-ABS-KEY ("Social Intelligence") AND TITLE-ABS-KEY ("Young Adults")) AND (LIMIT-TO (DOCTYPE, "ar"))
Timespan	1998:2023
Sources (Journals, Books, etc)	397
Documents	917
Annual Growth Rate %	12.5
Document Average Age	7.27
Average citations per doc	33.14
References	41972
Document contents	
Keywords plus (ID)	3603
Author's keywords (DE)	2000
Authors	
Authors	3350
Authors of single-authored docs	43
Authors collaboration	
Single-authored docs	46
Co-authors per doc	4.33
International co-authorships %	22.9
Document types	
Article	917

Annual Scientific Production

Between 1998 and 2023, the number of publications focusing on social intelligence and emotional intelligence in young adults has experienced fluctuations, with periods of both growth and decline. Notably, there was a significant increase in published works from 2008 to 2011, followed by a subsequent decrease. However, from 2017 to 2018, there was another upswing in publications. This cyclic pattern of alternating rises and falls is observable throughout the years. The highest number of publications occurred in 2018, with a total of 105 publications.

Most Significant Authors

The realm of social intelligence and emotional intelligence in young adults has seen the contributions of numerous authors, totaling 3350, whose published articles were taken as a gauge of their significance. Notably, Fernandez-Berrocal emerges as the primary contributor, boasting 18 published articles, closely followed by Petrides with 12 articles.

Most Relevant Sources

Our analysis of 917 papers gathered from 397 different journals indicates that the journal 'Emotion' exhibited the highest level of productivity, contributing an impressive total of 43 articles. Taking the second spot was the 'International Journal of Environmental Research and Public Health,' which published 37 papers. Following closely behind was 'Plos One' with 32 articles. Table 2 presents the top 10 journals that displayed the most significant output regarding social intelligence and emotional intelligence in young adults' research papers.

**Table 2:** The top 10 relevant sources.

Journal	Number of outputs
Emotion	43
International Journal of Environmental Research	37
Plos One	32
Psychiatry Research	19
Nurse Education Today	15
Cognition and Emotion	12
Psychological Reports	12
Journal of Personality Assessment	11
Personality and individual Differences	11
Psychology and Aging	11

Most Relevant Affiliations

Table 3 presents a visual representation of the key institutions actively involved in research on social intelligence and emotional intelligence in young adults. The University of Malaga stands out at the forefront, boasting the highest number of publications at an impressive 57. Following closely is the Harvard Medical School, which contributes significantly with 43 publications. Other notable contributors to this field of research include the University of California, the University of Melbourne, Tohoku University, the University of Almeria, the University of Arizona, the University of Western Ontario, Kings College London, and Trent University.

**Table 3:** Most relevant affiliations.

Affiliation	Number of outputs
University of Malaga	57
Harward Medical Scool	43
University of California	39
University of Malborne	33
Tohoku University	30
University of Almeria	26
University of Arizona	26
University of Western Ontario	26
Kings College London	24
Trent University	22

Three Field Plot of Keyword, Author, and Source

**Figure 1:**
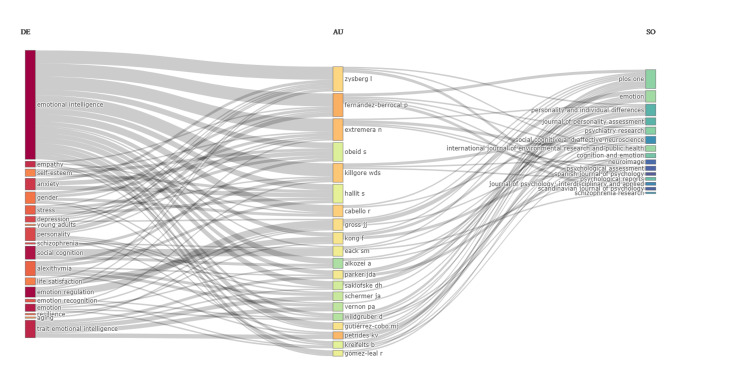
Three Field Plot, illustrating the correlation among author keywords (DE), authors (AU), and sources (SO). In the three-field plot, colours are used to represent additional layers of information or to differentiate between categories within each field. Keywords (DE): Colors associated with keywords indicate different sub-topics or themes within the research field. Authors (AU): Colors linked to authors signify their affiliations, the research group or institution they belong to. Sources (SO): The color coding of sources denote different publication.

The Sankey diagram in Figure 1 explores the relationship between keywords, authors, and sources within the context of social intelligence and emotional intelligence in young adults' literature. The purpose of the investigation was to identify frequently used keywords in the literature across various authors and published journals, suggesting a focus on understanding the role of emotional intelligence and social intelligence in young adults. The analysis of the top keywords revealed several prominent phrases frequently used in the literature. These include "emotional intelligence," "trait emotional intelligence," "emotion regulation," and "social cognition." The study found that specific authors were extensively involved in research related to emotional intelligence in young adults. Notable authors include Zysberg, Fernandez Berrocal, Extremera, Eack, and Schermer, among others. Their frequent use of the identified keywords suggests their expertise and contribution to this field of study. The investigation identified certain journals where research on emotional intelligence in young adults was frequently published. Some of these prominent sources include the journals Plos One, Emotion, and Personality and Individual Differences.

Co-occurrence of Keywords

**Figure 2:**
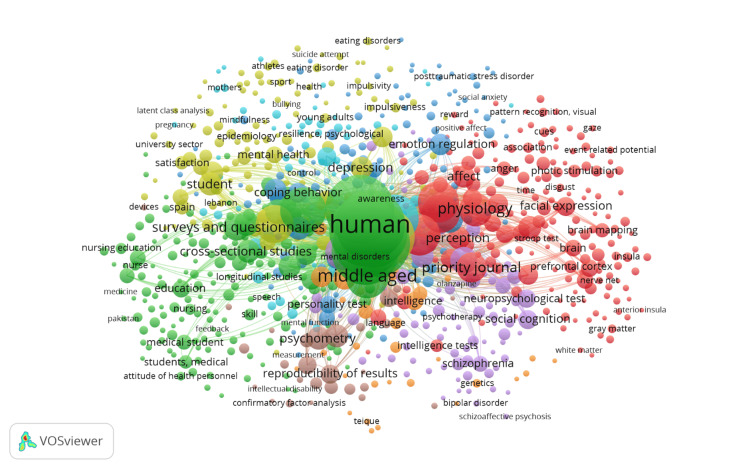
Visualizing the co-occurrence of all keywords using VOSviewer in a network format. The figure employs various colours to distinguish between eight different clusters. Lines are utilized to show the connections between each cluster. The colours themselves do not hold any specific meaning.

The VOSviewer software was employed to generate a visual representation of groups of keywords that appear together in the field of social intelligence and emotional intelligence in young adults. For this analysis, a subset of 805 keywords was chosen, all of which appeared at least five times out of a total of 5034 keywords. The findings are depicted in Figure 2. In this figure, the size of the nodes and the font used for each keyword depend on its weight value, which indicates its frequency of occurrence. Larger nodes and fonts are assigned to keywords that appear more frequently. The connections between nodes signify common co-occurrences between the keywords, with the thickness of the lines representing the strength of these co-occurrences. Thicker lines indicate a higher frequency of co-occurrence between two keywords. Upon analysing Figure 2, researchers identified eight distinct clusters. These clusters vary in size, with the first cluster containing 181 items, the second cluster having 170 items, the third cluster comprising 123 items, the fourth cluster including 122 items, the fifth cluster consisting of 86 items, the sixth cluster containing 64 items, the seventh cluster having 30 items, and the eighth cluster comprising 29 items. Among all the keywords, the term "human" appeared the most frequently, occurring 858 times, followed closely by "young adult" with 847 occurrences. The total link strengths (TLS) for these two keywords were 21,343 and 21,119, respectively.

Bibliographic Coupling of Sources

**Figure 3:**
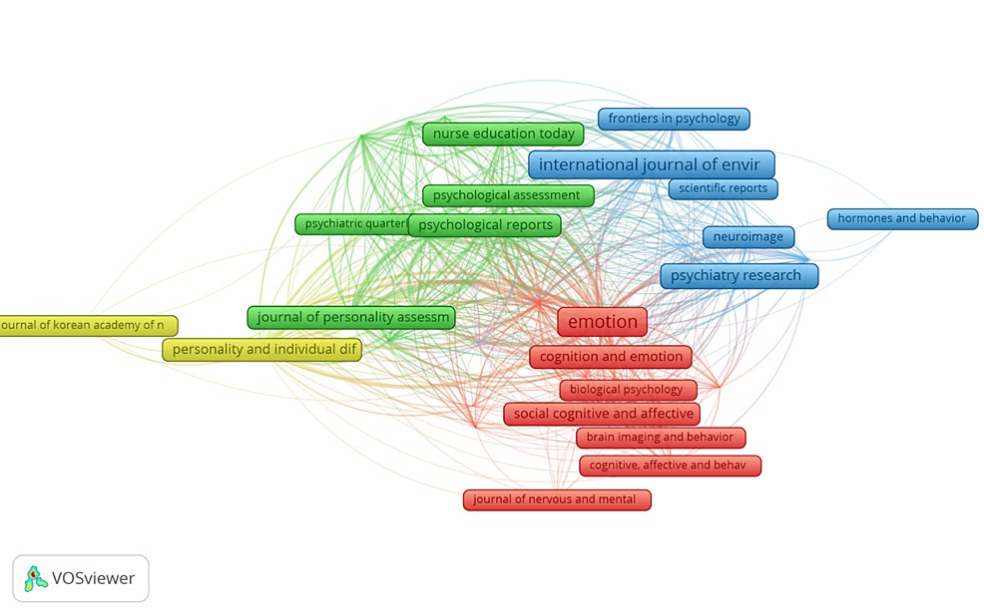
The network visualization of bibliographic coupling with sources. Different colours represent various clusters of publications: red signifies articles in the field of psychology, green denotes education, blue is used for medical-related publications, and yellow highlights those pertaining to personality.

The visual depiction shows a network that presents the connections between research articles related to social intelligence and emotional intelligence in young adults. Among the 397 sources that published these articles, only 37 were deemed suitable according to specific criteria. These criteria involved selecting sources with a minimum of five published articles and utilising a comprehensive counting method to evaluate their relevance. This network visualisation depicts the connections and interdependencies among various research articles and their respective sources. A comprehensive computation was performed to gauge the strength of the bibliographic coupling links within the 37 sources. The analysis identified a particularly significant TLS of 5066, which served as the basis for categorising the sources into five clusters, comprising a total of 37 items. The first two clusters consisted of 12 items each, while the third, fourth, and fifth clusters contained nine, three, and one item, respectively. Further examination of the data revealed that the most substantial combined link strength attained was 1257. This remarkable figure involved 43 articles, which collectively received 4337 citations from the journal "Emotion," earning it the top rank in this network mapping. Following closely was the journal "Plos One," securing the second position with 776 combined link strengths derived from 32 research articles. These results strongly suggest a notable collaborative effort between the two journals in publishing academic papers. Figure 3 is the visualisation of bibliographic coupling.

Countries' Collaboration World Map

**Figure 4:**
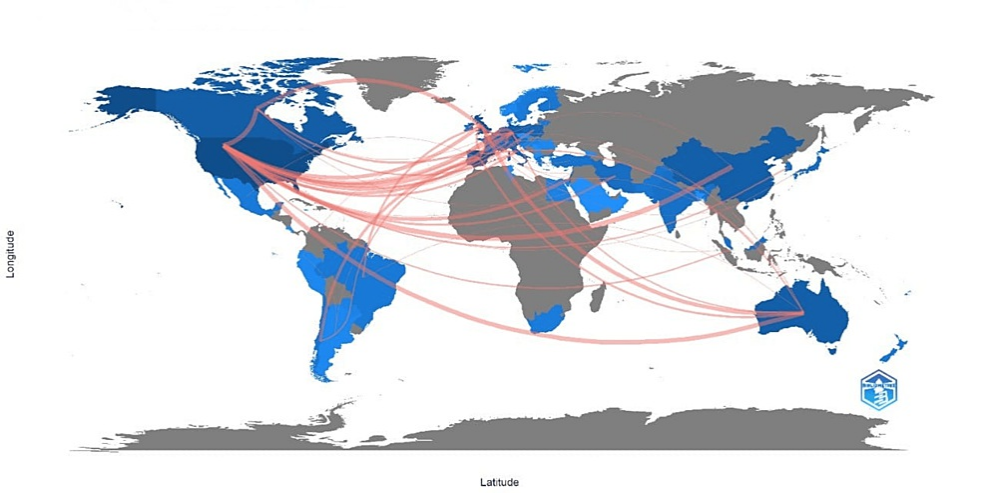
Countries' collaboration world map.

The information in Figure 4 reflects a thriving global research community focused on social intelligence and emotional intelligence in young adults. The use of blue to signify research cooperation among countries highlights the extent of global collaboration in this area. The United States of America stands out as the leading country in research collaborations in this field. The frequency of collaborations with China is exceptionally high, with 14 instances suggesting a solid partnership between these two countries in studying social and emotional intelligence in young adults. In addition to its collaboration with China, the United States actively engages in substantial partnerships with Canada (frequency of 12) and Australia (frequency of 11). This demonstrates the USA's commitment to fostering research relationships with these countries regarding social and emotional intelligence. The United Kingdom is also actively involved in research collaborations concerning social and emotional intelligence in young adults. It shows significant collaborative relationships with Australia (frequency of 9) and Canada (frequency of 9). Australia and Canada appear to be essential players in the global research network as they are involved in substantial collaborative efforts with the United States (Australia with a frequency of 11, Canada with a frequency of 12), and the United Kingdom (with a frequency of 9). As indicated by the visualisation, the extensive network of research cooperation among scientists on a global scale underscores the significance of studying social intelligence and emotional intelligence in young adults. Figure 4 is a depiction of the country's collaboration in this research area.

Discussion

The research employs articles from the Scopus database, spanning from 1990 to 2023, to analyse trends, collaborations, and thematic importance within the domains of emotional and social intelligence. Findings reveal an increasing scholarly interest in correlating these intelligences with critical personal and professional outcomes, including well-being and interpersonal success. The analysis highlights the role of prominent institutions and authors in advancing research, with thematic mappings showing significant ties between emotional intelligence and life skills such as self-esteem, empathy, and social aptitude. The study also underscores the growing acknowledgment of emotional and social intelligence as essential to young adults' holistic development, suggesting educational programmes integrate these concepts to enhance academic and life success. Future research directions include deeper integrative studies on these intelligences' educational impacts and their broader social implications.

The study provides a quantitatively enriched perspective on the progression and focal points of research in the area of emotional and social intelligence and the interplay of both in the life of a young adult. This study aligns with foundational theories by Salovey and Mayer [31], who originally proposed the term "emotional intelligence," and Goleman [32], who popularised it, affirming that these intelligences are critical abilities influencing personal success, professional achievements, and general well-being. This analysis corroborates earlier findings, as postulated by Brackett et al. [33], demonstrating a strong relationship between these intelligences and improved life outcomes such as academic success and workplace effectiveness.

Contrary to the narrow look at the influence of emotional and social intelligence on outcomes in academic and workplace setups, characterised by previous research [34], this bibliometric analysis perceives a wider scope of implications as being less emphasised in foundational models by Salovey and Mayer [31] and Goleman [32], ranging from those that pertain to well-being and holistic development. In a number of previous studies, these intelligences have normally been highlighted as separable or overlapping domains [35], while the present review will underline the convergent and strategic benefits of these intelligences in educational and policy-making frameworks [33]. The current findings, therefore, emphasise the importance of consolidating emotional and social intelligence for the fostering of communal well-being, thus marking a pivot towards the holistic approach in the understanding of young adult development, which hitherto has been dissimilar from the utilitarian focus in earlier works.

Practical implications

The study serves as a blueprint for the current scenario of research on this subject for those involved with emotional and social intelligence research among young adults, including researchers, educators, policymakers, and practitioners. It also maps key authors, journals, and institutions while identifying opportunities for collaboration and publication. These discussions would provide a comprehensive review of the field. Thematic mapping and keyword analysis can help discover emerging trends and guide further research and initiatives. This information is really invaluable for educators who include these findings in the curricula developed for enhanced, holistic development and well-being. Policymakers can also use such information to improve the condition of emotional health and encourage positive social interactions that would enable young adults to acquire the skills necessary for life.

Limitations

Despite its extensive utility, this review acknowledges certain limitations in its bibliometric approach. The exclusive use of the Scopus database may overlook significant studies present in other databases, potentially skewing the comprehensive understanding of the field. Furthermore, the inherently quantitative nature of bibliometric analyses typically excludes qualitative dimensions such as the depth and context of discussions found in individual studies. While the review efficiently identifies key contributors and maps the field’s dynamics, it does not evaluate the quality or the impact of the included studies, which could introduce biases related to self-citation and the varying citation practices across different scientific communities. These factors could influence the depicted trends and suggest areas for future research and collaboration, highlighting the need for a balanced approach that considers both quantitative and qualitative aspects of the literature.

## Conclusions

The study provides a thorough review of the evolution of the field of emotional and social intelligence among young adults. It seeks to achieve this goal through an analysis of articles indexed in the Scopus database. The analysis throws light on important trends, key contributors, and even some future sites of research and application in the context of education. The findings revealed the significance of these intelligences in personal and professional development and thus proposed that they be part of the curriculum for the improved balanced development and well-being of young adults. The review provides the researchers with information for further gaps to be explored. Future studies are recommended to explore deeper integrations of emotional and social intelligence with educational outcomes and wider impacts on society.
